# Hybrid Core-Shell (HyCoS) Nanoparticles produced by Complex Coacervation for Multimodal Applications

**DOI:** 10.1038/srep45121

**Published:** 2017-03-22

**Authors:** D. Vecchione, A. M. Grimaldi, E. Forte, Paolo Bevilacqua, P. A. Netti, E. Torino

**Affiliations:** 1 Istituto Italiano di Tecnologia, Center for Advanced Biomaterials for Health Care IIT@CRIB, Largo Barsanti e Matteucci 53, 80125, Naples, Italy; 2 University of Naples Federico II, Department of Chemical, Materials and Industrial Production Engineering, P.le Tecchio 80, 80125, Naples, Italy; 3 IRCSS SDN, Via E. Gianturco 113, 80143, Naples, Italy; 4 University of Naples Federico II, Interdisciplinary Research Center of Biomaterials, CRIB P.le Tecchio 80, 80125, Naples, Italy

## Abstract

Multimodal imaging probes can provide diagnostic information combining different imaging modalities. Nanoparticles (NPs) can contain two or more imaging tracers that allow several diagnostic techniques to be used simultaneously. In this work, a complex coacervation process to produce core-shell completely biocompatible polymeric nanoparticles (HyCoS) for multimodal imaging applications is described. Innovations on the traditional coacervation process are found in the control of the reaction temperature, allowing a speeding up of the reaction itself, and the production of a double-crosslinked system to improve the stability of the nanostructures in the presence of a clinically relevant contrast agent for MRI (Gd-DTPA). Through the control of the crosslinking behavior, an increase up to 6 times of the relaxometric properties of the Gd-DTPA is achieved. Furthermore, HyCoS can be loaded with a high amount of dye such as ATTO 633 or conjugated with a model dye such as FITC for *in vivo* optical imaging. The results show stable core-shell polymeric nanoparticles that can be used both for MRI and for optical applications allowing detection free from harmful radiation. Additionally, preliminary results about the possibility to trigger the release of a drug through a pH effect are reported.

Despite the recent advances in the single diagnostic techniques^1^, the study on diagnostic technology is still continuous, and it focuses on the disparity of diseases and the type of information provided by the single techniques^2^. In this scenario fits the Multimodal Imaging because it can combine two or more techniques based on different physical principles allowing the integration of functional information to structural ones^3^, overcome limitations of the single diagnostic techniques and optimize therapies leading to a “personalized medicine”^4^. Among the multimodal applications, Nuclear Magnetic Resonance-Optical dual imaging modality has the principle advantage to be completely free from ionization rays while anatomical and molecular, physiological or metabolic information are provided. Magnetic Resonance Imaging (MRI) is characterized by a relatively high spatial resolution, relatively no tissue penetrating limits but also it has a low sensitivity, high cost and long imaging time. Furthermore, recent concerns about the deposition of clinically relevant Gd-based Contrast Agents (CAs), linear and macrocyclic one are reported by Robert McDonald on neuroradiology^5^ and other publications^6^,^7^. Moreover, the recent opinion of the Food and Drug Administration (FDA) suggests the limiting the Gadolinium-based CAs use to clinical circumstances in which the additional information provided by the contrast is necessary. On the same course of action, a general review also has summarized the literature on gadolinium-based contrast agents^8^. We firmly believe in the importance to protect the Gadolinium, both linear and macrocyclic, and overcome the low specificity of the CAs. Furthermore, even if recent efforts have been done to improve the relaxivity of Gd-based contrast agents through the nanotechnologies, relaxivity is well below the required theoretical limits for linear and macrocyclic both, and the boost of the signal is still a valuable issue^9^,^10^,^11^,^12^.

Differently from MRI, Optical Imaging has a higher sensitivity; it is a multicolor Imaging which allows visualizing target biological molecules (peptides, drugs, etc.) but it presents a low spatial resolution and a poor tissue penetration. Among different optical techniques, Fluorescence Molecular Tomography (FMT) produces quantified 3D reconstructions of fluorescence activation or concentration. In this framework, their combination can provide information not available typically by applying the single methodologies and allows to distinguish finely in different tissues within the body^13^. The introduction of nanotechnology has led to the development of many medical products including the formulation of new devices that can be used for Multimodal applications^13^,^14^,^15^,^16^,^17^. Among them, nanovectors can potentially provide early diagnosis and monitoring of therapeutic response^18^,^19^ and play a significant role in the dawning era of personalized medicine^20^,^21^,^22^. Nowadays, although many efforts are made in the development of instrumentations for multimodal imaging, this field still lacks biocompatible, biodegradable and FDA approved probes^23^. In literature, different studies about dual imaging MRI and Optical Imaging are reported^16^,^24^,^25^. In 2009, Xie *et al*.^26^ produced Iron oxide nanoparticles (IONPs) for Positron Emission Tomography(PET)/Near InfraRed Fluorescence(NIRF)/MRI applications labeled with Cy5.5 dye and ^64^Cu-DOTA chelates. In the same period, Chen *et al*.^27^ presented the IONPs nanoparticles coated with a PEGylated amphiphilic triblock copolymer conjugated with a Near-Infrared Fluorescent (NIRF) dye IR-Dye800 and with 64Cu-DOTA chelates. In 2010, Nam *et al*.^28^ reported tumor targeting nanoparticles for optical/MRI dual imaging based on self-assembled Glycol Chitosan (GC) which is chemically modified with 5β-Cholanic Acid (CA). For optical imaging, (GC-CA) is used as a chelate for Gd(III) and Cy5.5 is conjugated. Tan *et al*.^29^ in 2011, presented a multimodal imaging system by co-encapsulating superparamagnetic iron oxides (IOs) and Quantum Dots (QDs) in Poly(lactic acid)-d-αtocopheryl polyethylene glycol 1000 succinate (PLA-TPGS). Then, in 2013 Huang *et al*.^30^ produced Mn_3_[Co(CN)_6_]^2^ nanocubes that can serve as MRI agents and Two-Photon Fluorescence (TPF). Tian *et al*. in 2013, reported core-shell Fe_3_O_4_-Cu_9_S_8_ nanoparticles for dual-modal imaging and photothermal therapy^31^, useful as a probe for T_2_. Among these different probes, those combining MRI and Fluorescence Molecular Tomography (FMT) imaging modalities have mainly been used to evaluate morphological and functional changes in tumors in response to chemotherapy by monitoring tumor growth and protease activity^32^. Here, we aim to design a probe that can combine multimodal properties to improve and facilitate the use of integrated imaging modalities. The probe is also completely biocompatible and realized with FDA approved materials to promote the transition of results to preclinical and clinical Multimodal Imaging. The design of the probe is achieved through an intriguing modification of the traditional complex coacervation able to create a stable architecture and to provide impressive MRI performances and release behavior. The coacervation has been less explored for diagnostic applications due to its complexity although it appears to be a highly productive method^14^. Among different methods of production, we have selected the Emulsion-Coacervation process because of its reliability in obtaining core-shell nanosystems^33^. Indeed, several advantages are the enormous flexibility due to the numerous parameters to modify for the proper success of the reaction such as pH, temperature, ionic strength, polyacid/polybase ratio, polymer concentration, the molecular weight of polyelectrolytes able to control the thickness of the layer of coacervate and the polydispersity index^34^,^35^. Furthermore, the choice of a core/shell architectures allows the obtaining of highly functional materials^36^ with modified properties by changing either the ratio of the constituting materials or the core to shell size. Among several advantages offered by this approach, the translation of the process on a large scale, the high productivity and the high flexibility of the system are the most attractive. Currently, nanoparticles obtained by coacervation for applications in MRI as contrast medium has been evaluated to allow the increase of the signal encapsulating substances such as Gd chelates, whose free molecule is toxic into the body^25^. To date, most of the efforts have been limited to grafting contrast-enhancing agents to the surface of hydrophobic particles, where access to water is achievable or to enhance the delivery by and effective encapsulation. However, exploitation of the role of coacervation and the possibility of the Emulsion based Coacervation processes to boost T1 in combination with optical imaging has not been performed^37^,^38^. Our fundamental understanding of the Complex Coacervation Method is applied to the production of Hybrid Core-Shell NanoParticles (HyCoS NPs) made of a Chitosan-core and a Hyaluronic Acid-shell, and to the control of their properties for application in the MRI and Optical Imaging Field. Indeed, HyCoS NPs are designed to entrap rationally, a relevant clinical CA, Gd-DTPA, enhancing its MRI performances and delivering a high amount of an optical tracer at the high-intensity signal. These nanovectors are engineered to own these peculiar properties and, at the same time, preserve fundamental characteristic of the delivery (e.g. size, charge, choice of shape and materials)^39^,^40^. Furthermore, our efforts are made to overcome the interference of MRI agent with the coacervation process introducing a double crosslinking reaction, an intrachain crosslinking (between polymer chains of core-shell) and an interchains one (between the polymer chains of the shell), able to improve the stability of the system. Then, using the temperature, the proposed process has been speeded up to reduce the reaction time and stabilize the architectures.

## Results

### Phase behavior of the Ch-AcOH-Water system

**Figure 1:**
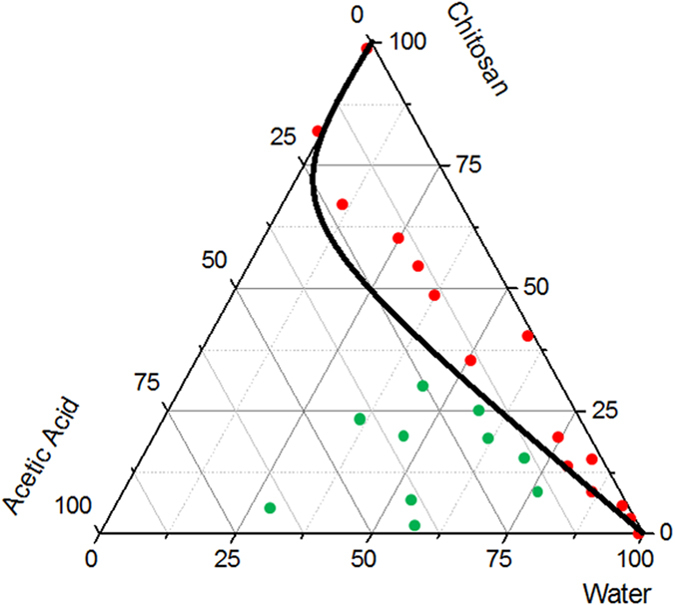
Experimental determination of the Phase Diagram of the ternary system Water-Acetic Acid-Chitosan. The Phase Diagram is obtained adding dropwise the acetic acid to the Chitosan and Water, allowing the complete dissolution of the polymer. The green spots (-**•**-) represent the working point showing the complete dissolution of the polymer. The line drafts the miscibility gap between the two different behaviors.

Our first step is devoted to the understanding of the phase diagram of the ternary system Water-AcOH –Ch (Fig. 1) to improve the knowledge and, therefore, the stability of the aqueous phase of the emulsion template. The thermodynamic system is extremely complex and sensitive to small changes in concentration among the components. The low solubility of the Ch creates a gap of miscibility that is overcome by adding the AcOH dropwise. The ternary diagram, reported in Fig. 1, is built analyzing the behavior of Ch at different concentrations of the AcOH solution. The red working points show the concentrations at which the polymer is not dissolved in the solution while the green working points display the complete dissolution of the polymer chains. The black line, which draws the border of miscibility gap, shows a scratched transition area between the two different behaviors. For a concentration of 1%wt/v, the complete dissolution of Ch in 5 mL of water is reached only at 2 μl/ml of AcOH.

### Preparation of the Hybrid Core-Shell Nanoparticles (HyCoS NPs)

**Figure 2:**
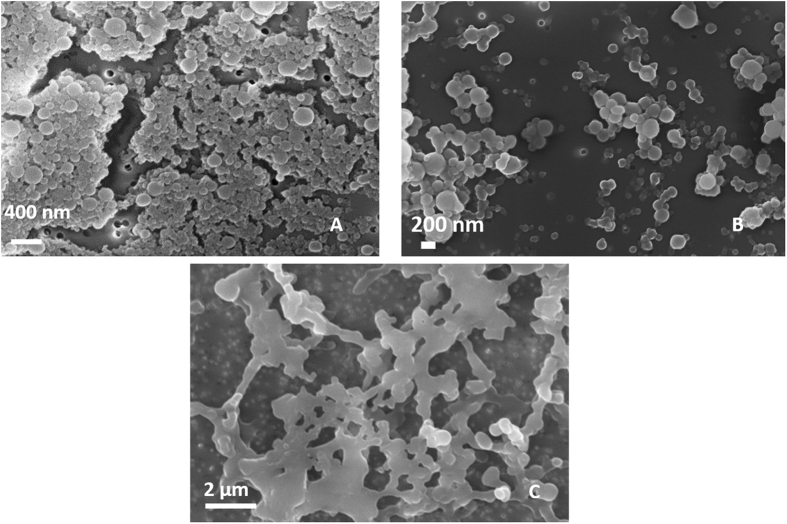
Morphological characterization of nanoparticles by Fe-SEM. Coacervation performed at 0,1% wt/v HA, 1% wt/v Chitosan, 30% TPP wt/v, Gd-DTPA:Chitosan 1:1. (**A**) and (**B**) NPs collected in EtOH while (**C**) NPs collected in water. Results clearly show that if Gd_DTPA is added to the solutions, stability of the CoA NPs in water is compromised and the spherical core-shell morphologies are not preserved.

**Figure 3:**
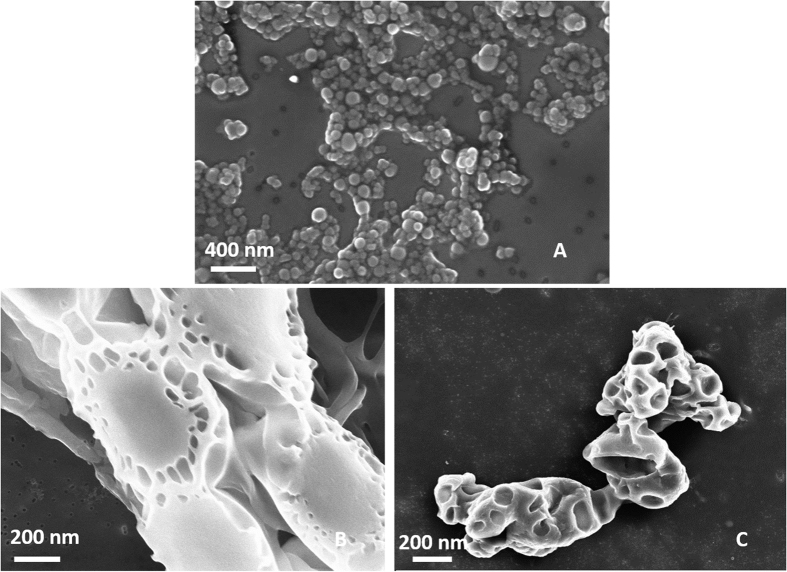
Morphological characterization of nanoparticles by FE-SEM. Coacervation performed at 0,1% wt/v HA, 1% wt/v Chitosan, 30% TPP wt/v, Gd-DTPA: Chitosan 1:1. NPs collected in water obtained by using DVS at (**A**) 8% v/v; (**B**) 16% v/v; (**C**) 24% v/v DVS collected in water. Panel reports how the stability of the c ore shell nanovectors is reached only for DVS at 8% v/v.

Nanoparticles are produced by coacervation performed at 0,1% wt/v HA, 1% wt/v Ch, 30% wt/v TPP, showing a stable behavior also in water. However, when Gd-DTPA is added to the aqueous phase at the ratio 1:1 GdCA:Ch, morphologies of the nanoparticles results irreversibly compromised in water (Fig. 2). Indeed, even if the coacervation is properly occurred in the presence of Gd-DTPA, as observed in Fig. 2A and B reporting nanoparticles in EtOH, the spherical shape is not preserved in water and dissolved completely just after few minutes. This evidence shows a strong interference of Gd-DTPA with the crosslinking reaction that weakens the effectiveness of the coacervation. To avoid the instability of the architectures in the presence of Gd-DTPA, we have proposed the introduction of a second crosslinker, DVS able to create bis-sulfonyl linkage between OH groups of HA^41^,^42^. Adding the different percentage of DVS (8%, 16%, 24% v/v), it is noticed that there are also some regular swelling phenomena by increasing the volume of DVS for NPs with and without Gd-DTPA (Fig. 3A–C). Indeed, NPs maintain their structures, avoiding the whole dissolving phenomena only at DVS concentration of 8%v/v (Fig. 3A). At percentage of DVS higher than 8%v/v, (Fig. 3B and C) there is an increase in the size of the nanoparticles and the production of some irregular shapes. This effect is probably due to interference between DVS and the system caused by a partial reaction of the crosslinker with the hydroxyl –OH groups of HA (Fig. 3B and C).

### FT-IR analysis

An FT-IR analysis is performed to confirm the effectiveness of the chemical reaction. Indeed, the favorable pH conditions reported in the literature for HA and DVS^41^ are not properly reached in our system, and an FT-IR study is conducted to investigate the interaction between the two polymers (Ch and HA) and the crosslinkers (TPP and DVS). In Figure S1 of the Supporting Information spectra of Ch-HA nanoparticles is shown. A band at 3450 cm^−1^ that can be attributed to –NH_2_ and –OH group stretching vibration in chitosan matrix is currently not detected in the spectra of HyCoS NPs particles^34^. The characteristic bands at 1658.64 cm^−1^ and 1730.92 cm^−1^ is attributed to C=O group stretching vibration in the HA matrix. A new sharp peak 1641 cm^−1^ has emerged, and the 1585 cm^−1^ peak of –NH_2_ bending vibration has shifted to 1582 cm^−1^. Because of –NH_2_ bending vibration shifts, the features bands at 1614 cm^−1^ and 1405 cm^−1^ have shifted to 1641 cm^−1^ and 1377 cm^−1^ respectively. The presentation of P=O vibration absorption at 1269 cm^−1^ is observed, indicating the reaction between Ch and TPP. The peaks at 2953.42 cm^−1^, 2922.48 cm^−1^ and 2853.39 cm^−1^ show the −CH interaction. The characteristic peaks for DVS show absorptions at 1119.76 cm^−1^ (S=O symmetric stretching vibrations) and 720.99 cm^−1^ (S–C stretching vibrations) and through the ether bond at 1269 cm^−1^ (C–O–C stretching vibrations). The DVS has an effect on the –OH groups of the HA chains and the favourable condition for this pH reaction is between 10 and 12. Furthermore, the linking between the aminic groups of Ch and the phosphoric groups of TPP and the –COOH groups of HA is not well promoted due to the strongly acid conditions of the chitosan-water phase. The acid state of the Ch phase is essential not only for the complete dissolution of the Ch in the AcOH solution but also for the behavior of the Ch chains. In fact, at the strongly acid condition, even if more groups are activated, they are set in a shape not favorable to the interaction with the TPP. On the contrary, at weak acid conditions, even if there are less free amine groups, they are set in a more favourable shape that allows them to interact with TPP through the 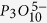. To improve the stability of the polymeric NPs different percentage of the two crosslinkers are tested. As previously shown 10%, 20% and 30% wt/v are tested for TPP while 8%, 16%, 24% v/v are tested for DVS. The results show not only a different stability of NPs in water but also a variation in their size. Increasing the TPP volume from 10% wt/v to 30% wt/v, the stability of the NPs is improved, and, at the same time, the size of the NPs decreases. The effect obtained in the case of DVS is completely different. In fact, an increasing of DVS percentage results in both an increasing of the size and the development of irregular shapes. It highlights the difficult of DVS to spread through the emulsion in the presence of a high concentrated oil phase. DVS concentration of 8% v/v and TPP concentration of 30%wt/v produce the stabilization of HyCoS NPs with a size in a range from 50 nm to 200 nm preserving also the coacervation process. It is important to note that the percentage of TPP is also increased from 20% to 30% wt/v in the HA phase (pH 10–11) to reach the final pH of the reaction (pH 5). In these conditions, the final values of pH is about 5, making stronger the linker between the two polymers. For the final reaction at pH value lower than 5, the crosslinking reaction of TPP between Ch and HA is not completed because the –COOH groups of HA are not fully activated while at pH higher than 5, available amine groups of Ch to the reaction are reduced.

### Effect of the Temperature on the coacervation step

**Figure 4:**
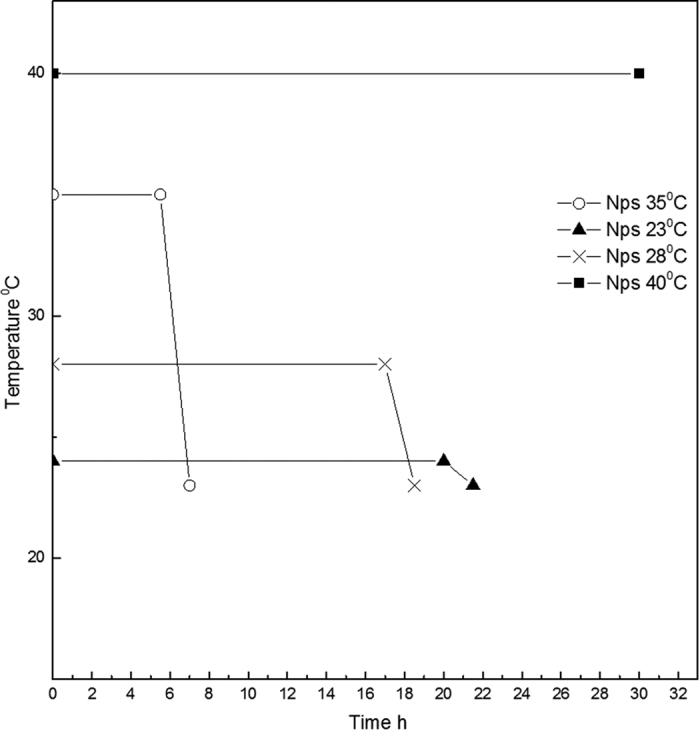
Demixing profile of the Reaction. In the graph is shown the profile of demixing of the reaction at different gradients of temperature. Temperature induces a reduction of the process time from 24 h to 6.5–7 h. Best results are obtained performing an Isothermal at 35 °C for 5.5 h and a ramp from 35 °C to 23 °C for 1.5 h. In details, after the isothermal step, the reaction of coacervation occurs, and a demixing of the prepared solution is observed. After the demixing phase, a variation of the temperature should be suddenly performed to avoid aggregation phenomena and the destruction of the nanovectors. Indeed, as soon as the demixing phase occurs, the produced nanoparticles are unstable and a further support of temperature needed to increase the stability of the system.

**Figure 5:**
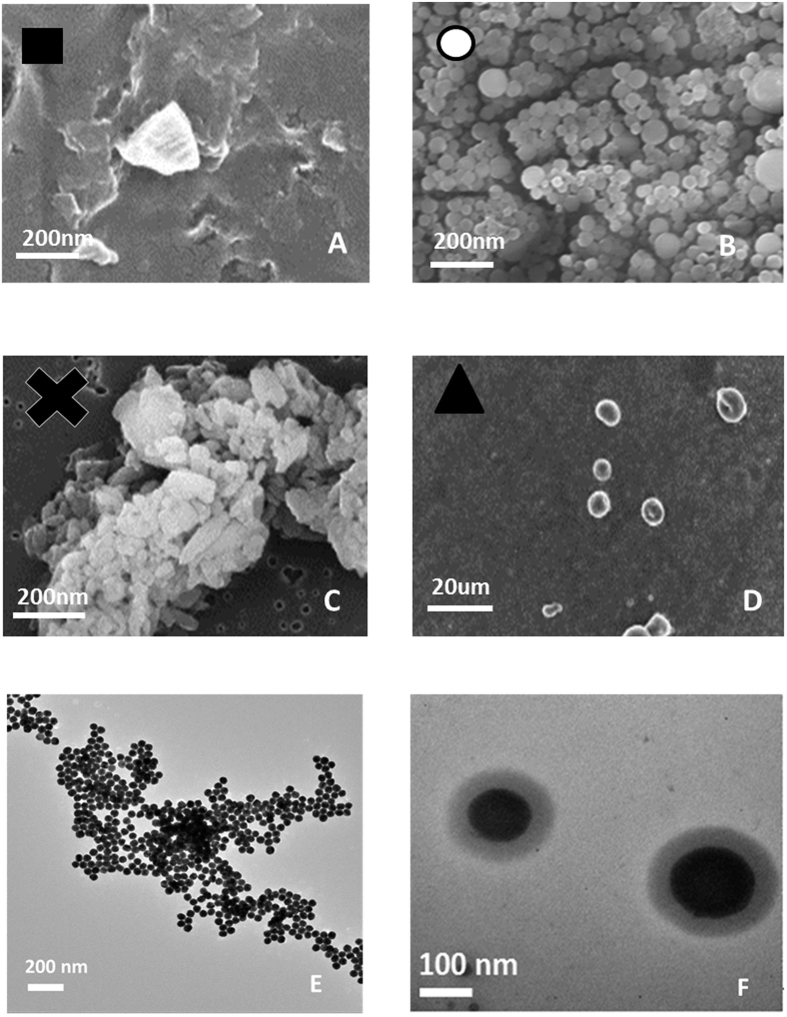
SEM images of nanoparticles produced by different calorimetric profile (**A**–**D**). A (**•**)- Nps obtained performing an Isothermal at 35 °C for 5.5 h; B (□)- Result obtained performing an Isothermal at 40 °C. No demixing is observed; C (Х)- Result obtained performing an Isothermal at 28 °C until demixing (after 17 h). D (**▴**)-Result obtained performing an Isothermal at 23 °C until demixing (after 20 h). After that the demixing occurs, a ramp to 23 °C for 1.5 h is performed to complete the separation and to stabilize the morphologies. Morphological characterization of nanoparticles by TEM (**E–F**). Core-shell Gd-DTPA-Nps obtained without the use of the temperature profile collected in water. (**E**) Nps obtained at 0,1% wt/v HA, 1% wt/v Chitosan, 30% TPP wt/v, Gd-DTPA: Chitosan 1:1 and 8% v/v DVS and (**F**) Magnification of the previous image.

Furthermore, to minimize the time of the coacervation step, different gradients of temperature are tested (Fig. 4). The intent is to promote both the evaporation of the AcOH and the chemical reaction of *inter* and *intra* chains. Several isothermal profiles are studied but the formation of nanoparticles is only observed at 35 °C, confirming that the coacervation step correctly occurs even if the morphologies are not stable in water, probably due to an uncompleted chemical reaction among the compounds. Later, starting with the isothermal observations, we have studied the combination of an isothermal profile with a cooling step. In this experiment, the demixing is visible after 5,5 h of isothermic step at 35 °C followed by a ramp at 8 °C/h from 35 °C to 23 °C, spending about 1.5 h to reach the final stable morphologies. It is important to note that, after the isothermal step, when the demixing visibly occurs, the cooling of the emulsion should be suddenly performed to avoid aggregation phenomena among the nanovectors. Indeed, the additional cooling step is needed to increase the stability of the system. A reduction of the demixing time involves a decreasing in the evaporation time of the AcOH, a faster coacervation reaction and an acceleration of the DVS reaction with the –OH groups on the polymer chain of HA. The use of temperature allows a reduction of the response times from 24 h to 6,5–7 h and it also allows a comparison regarding swelling behavior with HyCoS NPs obtained at constant room Temperature, as reported in the next paragraphs. Images reported in Fig. 5 show nanoparticles obtained at the different demixing profile of the reaction at different temperatures. NPs obtained performing an Isothermal at 35 °C for 5.5 h show the best stability and a defined core-shell morphology (Fig. 5 -**•**-). In Fig. 5E, a morphological characterization of core-shell nanoparticles obtained by TEM is shown. In details, several nanoparticles of 70 nm produced by our standard coacervation are reported while, in Fig. 5F, an enlargement is displayed highlighting the entire core -shell architecture. Additional TEM images are presented in the supporting information (Figure S2) and a 3D representation obtained by a cryo-TEM Tomography is also available.

To assess the formation of the Core-Shell Structure, Zeta Potential measurements are also performed to support the morphological characterization provided by TEM images and verify the deposition of HA on the positively charged Chitosan Core. Results regularly show −45 mV (Standard Deviation ± 5 mV) on the slightly positive surface charge of the Chitosan core alone (+15 mV). HyCoS NPs are negatively charged due to the deposition of HA on the outer face of the Chitosan core.

### pH-sensitive behavior

Preliminary results on HyCoS NPs report a peculiar yet interesting pH-sensitive behavior of the nanovectors. In details, HyCoS NPs produced at room Temperature, at pH 4, maintain their stability until the first hour, but after this time they dissolve completely. On the contrary, at pH 7 they preserve their stability at all the different times (30 min, 1 h, 2 h, 3 h, 6 h, 8 h, 12 h, 24 h). Nanoparticles obtained by using the cooling step do not seem to be pH-sensitive and remain stable at any pH condition until 24 hr. Further details are reported in Figure S3 of the Supporting Information, showing images of the different behavior of the NPs by changing the pH conditions.

It is well known that the HA- DVS crosslinking is basic pH-sensitive due to the ionization of the carboxyl group(–COOH) gives rise to anionic carboxylate(CH3COO–). Consequently, the electrostatic repulsion between the negatively charged groups results in dramatic swelling behavior.

Therefore, the pH-sensitive behavior can be only attributed to the swelling of the coacervation layer linked by anions and –COOH- that it has firstly formed on the surface of the Ch droplets.

This peculiar pH triggering could provide significant advances in the use of the vector for the treatment of tumor pathologies. In fact, the tumor environment, besides to being devoid of oxygen, is also characterized by an acid pH by an impaired operation of the lymphatic system to drainage of fluids that instead tend to accumulate inside the interstitial space^39^,^43^,^44^,^45^. First, the use of macromolecules and, later, nanocarriers is exploited to study the potential acidity of the tumor microenvironment to release any drugs encapsulated in the carriers directly in the tumor area to have a diffusion of the active agents within the tumor matrix and, therefore, performed a better diagnosis or therapy. In conclusion, the disruption of the NPs inside the cell greatly influences the resulting toxic effects. This cytotoxicity can be desired, in antitumor treatments, or more or less undesired during NPs application for diagnostics purposes. Furthermore, an excellent cell uptake of the NPs guarantees the efficient cell MRI and intracellular drug delivery.

### *In vitro* MRI

**Figure 6:**
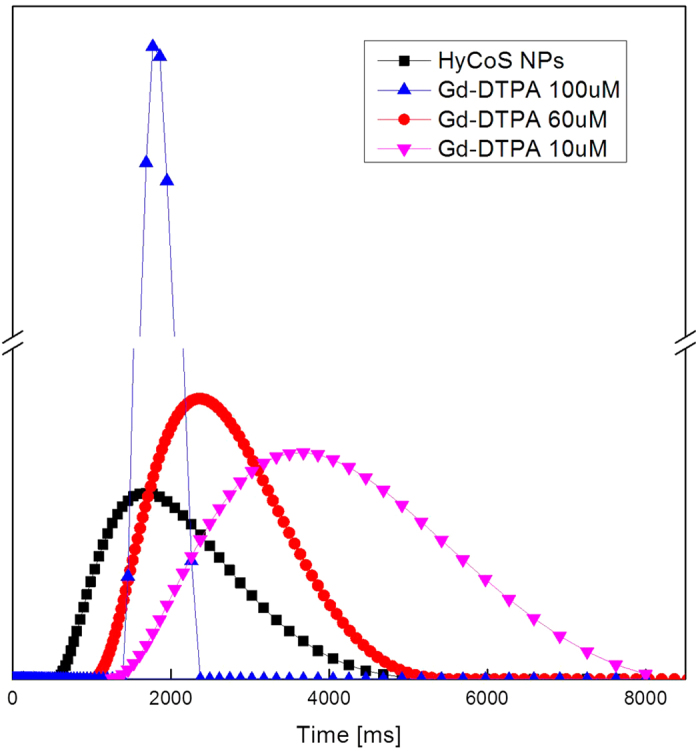
*In vitro* MRI. Relaxation rate distributions of: Gd-DTPA in solution at (-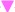-)10 μM; 60 μM (-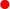-), 100 μM (-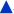-); and HyCoS NPs loaded with Gd-DTPA at (-■-)The results show an enhancement in r_1_ of 6 times compared to the free Gd-DTPA. T1.

As already reported in the Materials and Method Section, ICP-MS is used to assess the concentration fo Gd-DTPA loading within the HyCoS NPs. This data are also useful to evaluate the relaxometric properties of the nanovectors. Results clearly demonstrate that a relaxation rate T_1_ of 1720 ms is achieved with 20 μM of Gd-loaded HyCoS while 100 μM of Gd-DTPA solution is required to reach similar T_1_ (1724 ms) (Fig. 6). This effect of enhanced relaxivity potentially allows the administration of a reduced dosage of contrast medium keeping the same T_1_ signal intensity. This peculiar behavior is probably ascribable to the combination between the hydrophobic nature of the Ch and the hydrophilic character of the HA tuning the water exchange with the coordination sites of the metal chelates.

### Optical Imaging and bioconjugation

Two strategies are approached to evaluate the optical imaging properties of the HyCoS NPs: loading of the dye within the shell and bioconjugation of the dye to the surface of the HyCoS NPs.

**Figure 7:**
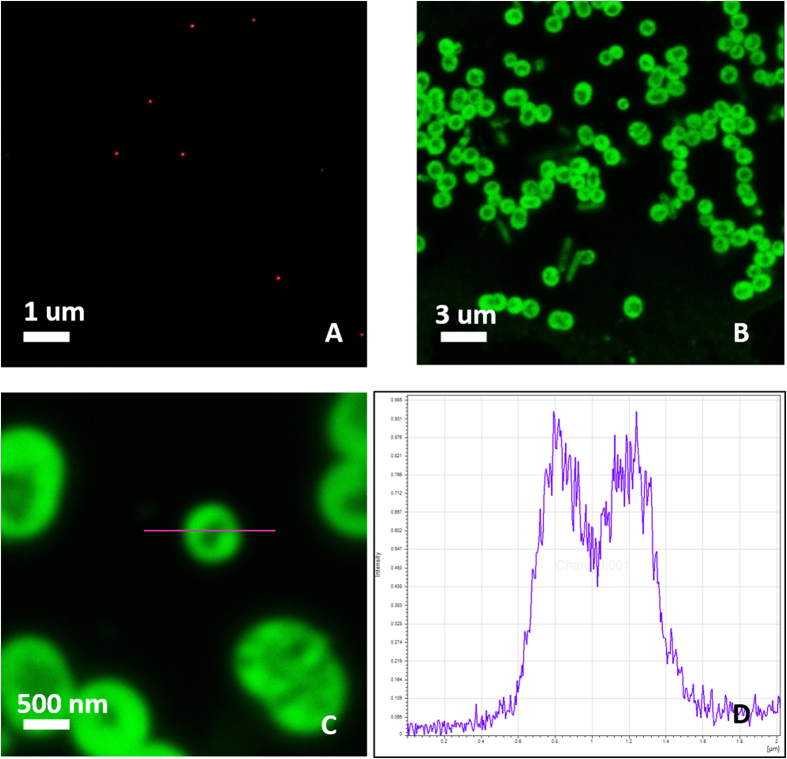
Optical Imaging of the nanoparticles by STED. NPs obtained at 0,1% wt/v HA, 1% wt/v Chitosan, 30% TPP wt/v, Gd-DTPA: Chitosan 1:1, 8% v/v DVS and Gd-DTPA: (**A**) Gd-DTPA and 100 μl of Cy5 1 mg/ml (633 nm) are encapsulated into the NPs; (**B**) The obtained nanoparticles are conjugated with FITC (488 nm). The image shows the difference between the core and the shell structure; (**C**) Magnification of the image reported in figure (**B**), (**D**) intensity profile of fluorescent Nanoparticle in (**B**) calculated by the Region of Interest method (ROI).

In Fig. 7, results related to the optical imaging of the nanoparticles observed under STED are reported. The first image (Fig. 7A) shows nanoparticles obtained by coacervation performed at 0,1% wt/v HA, 1% wt/v Ch, 30% TPP wt/v, Gd-DTPA: Ch 1:1, 8% v/v DVS.

Cy5 ranging from 0.1 to 1 mg/mL (633 nm) is encapsulated into the NPs while, in Fig. 7B and C, the NPs are bioconjugated with FITC (488 nm) via EDC/NHS reaction. In (Fig. 7D), an intensity profile of the conjugated NPs of Fig. 7C is reported. Figure 7B and C report the fluorescence due to the conjugation of the dye exclusively on the shell, highlighting the non-fluorescent core not involved in the reaction. However, while the Cy5- loaded HyCoS NPs preserve their size and shape (Fig. 7A), an increase of the shell size is observed for the FITC-bioconjugated HyCoS NPs. Due to these results, we have also checked that HyCoS NPs are capable of preserving their cargo of Gd-DTPA as so their boosted relaxometric properties. This behavior is probably due to the interference of the chemical reaction with the external shell, keeping intact the chitosan core containing the Gd-DTPA. It is an important goal considering the relevance to candidate our product for the *in vivo* Integrated Multimodal Imaging.

### *In vitro* cytotoxicity

Typically, structural alterations of NPs in aqueous solutions, in cell-culture medium, might also affect and change the final results of the *in vitro* toxicological studies. Therefore, Cytotoxicity tests are essential to assess preliminarily the biocompatibility of the HyCoS NPs. Nasti *et al*.^46^ suggest the cytotoxicity of the chitosan/TPP nanoparticle to be mostly dependent on their internalisation, which on its turn seems to be scarcely dependent on size and clearly dominated by surface composition/charge: indeed it is well known that positively charged nanoparticles are more quickly internalised than negatively charged ones and the HA-coating markedly reduces the nanoparticle toxicity. Results of chemical characterization studies show that the amount of covalently incorporated DVS into the structure of HA is largely controlled by the crosslinker concentration, thereby determining the mechanical stability and resistance against enzymatic degradation. Lai demonstrates good cytocompatibility of HA sheets treated with concentrations of DVS ranging from 0 to 50 mM^47^,^48^. For this study, the toxicity of HyCoS NPs is tested using a WST-1 assay, which is based on the conversion of a water-soluble tetrazolium salt (yellowish in color) to water insoluble formazan (purple color) by living cells. The WST assays have appeared to be advantageous over MTT because of its solubility in tissue culture medium and storage condition.

**Figure 8:**
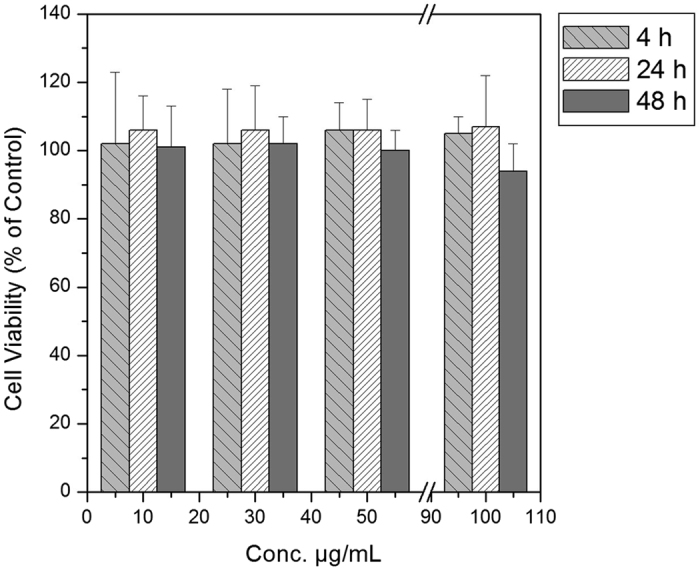
*In vitro* cytotoxicity. A549 cell viability expressed as a percentage of the value obtained with a concentration of hyaluronic nanoparticles in a range between 10 μg/mL and 100 μg/mL for three different time intervals. The error bars represent the standard deviations calculated from three independent experiments.

Scalar concentrations of HyCoS NPs are tested in a range between 200 ng/mL and 100 μg/mL. It is evidenced that the NPs showed no detectable cytotoxicity *in vitro* (Fig. 8).

### Successful conditions to produce HyCoS Nanoparticles with Enhanced Multimodal properties

**Figure 9:**
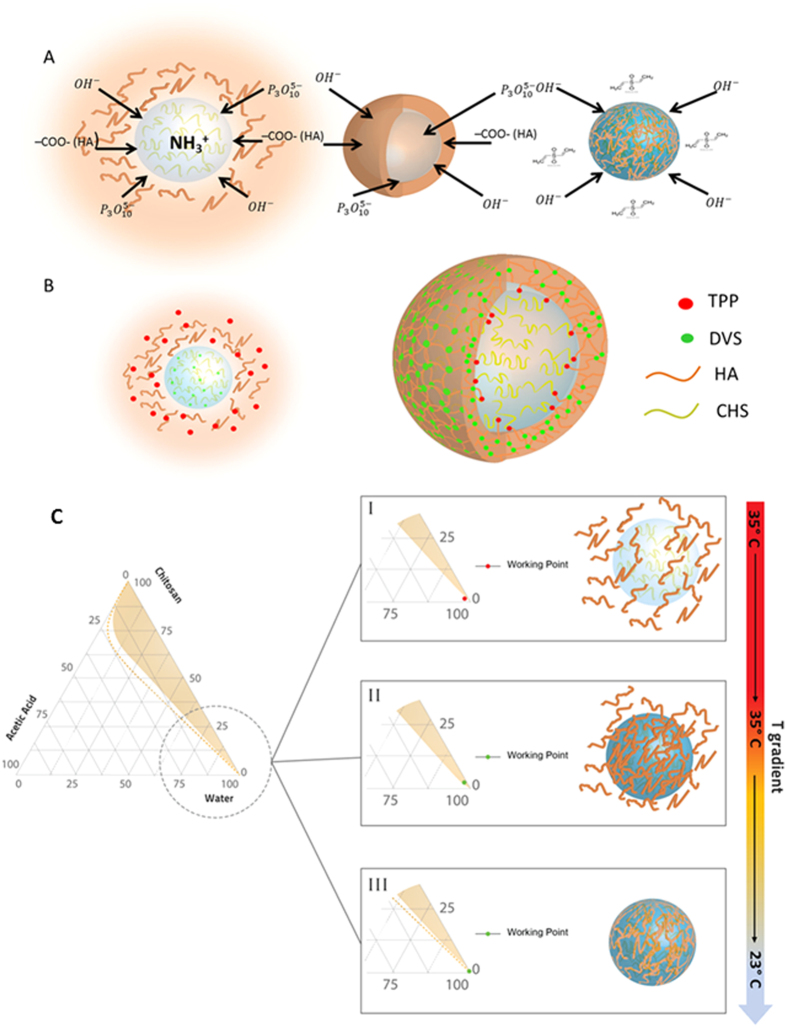
Description of Double Crosslinking Reaction. (**A**) On the left, the ionic interaction between TPP and Chitosan is described; later a crosslinking between Chitosan and HA is created through the TPP, on the right, in the last step the crosslinking between HA and DVS is obtained. (**B**) Diffusion and formation of double-crosslinked core-shell. Schematical representation of the crosslinking at the interface between the core and shell structure. (**C**) A schematical explanation of the process by the phase diagram change. Starting by the considerations reported in Fig. 3 about the reactive groups, this representation reports details about the thermodynamic involved in the proposed coacervation process. **I**- After the preparation of the primary emulsion, disperse phase contains a homogeneous solution. Therefore, the working point represented in the phase diagram is located in the region of complete miscibility between water and Chitosan. **II**- Solution containing the coacervate is added to the primary emulsion, promoting the dilution of the disperse phase, promoting the supersaturation of the solute in the disperse phase and inducing the precipitation of the chitosan. At the same time keeping constant the temperature, Acetic acid continues to evaporate producing the further shifting of the working point into the miscibility gap. This last step will complete the formation of the chitosan template and the HA coacervation; **III**- In the final phase, a controlled cooling step is performed to increase the miscibility gap able to promote a faster supersaturation and to enhance the stability of the nanoparticle’s architecture.

The described process has led to the generation of NPs with a Chitosan core and an outer shell of HA. The innovations of process consist of a double crosslinking, by TPP and DVS, which allows controlling the stability and the degradation behavior of the two chosen polymers, and a heating/cooling step of the reaction to speed up the coacervation process and control the swelling behavior. TPP is considered as a small ion with a triple negative charge which is dissolved into the HA phase, able to link the hydrophobic core of Ch to the hydrophilic shell of HA. On the HA side, –OH(HA) and –COOH (HA) can coexist in the tripolyphosphate solution at all pH values promoting the interchains reaction between the two polymers. Indeed, when the solution containing the HA and the TPP reaches the surface of the droplet template containing Ch and DVS, a coacervation layer has firstly formed on the surface of the Ch droplets, and anions and COOH (HA) reacts with caution. Simultaneously, the –OH groups of HA are linked by DVS, stabilizing the shell. (Fig. 9A), while TPP partially diffuses inside the template through the coacervation layer reaching the core and creating hydrogen bonding, linking the Ch core to the layer of the HA firmly (Fig. 9B). However, because HA has a molecular weight larger than the Ch, and Ch is a hydrophobic polymer, both HA and TPP cannot deeply penetrate the core, probably reaching only the surface of the Ch template. The above-reported steps are here described for the first time and represent a significant advance in the understanding of the control of the interference due to the presence of Gd-DTPA in the crosslinking reaction and the coacervation process. A unique aspect of this system also lies in the double crosslinking promoting both an intrachain link among HA polymer chains and interchain links among Ch and HA able to control the loading of Gd-DTPA and its relaxometric properties. Furthermore, the TPP is added in HA coacervate phase while in literature is typically added to the chitosan phase. In details, phosphoric groups of TPP interact with the amine groups of Ch and with –COOH and –OH groups of HA, while sulfuric groups of DVS links –OH groups of HA. The reverse use of the two crosslinkers is fundamental to avoid the linkage between the chains of HA before the coacervation reaction occurs. In fact, the DVS is not able to interact with the Chitosan chains while TPP can link HA chains only partially^46^. The pH of the single polymeric solutions is deeply studied to control entirely the reverse use of crosslinkers. Indeed, the pH of the Ch and HA phase are controlled to allow the activity of the crosslinkers only during the coacervation process and not during the preparation of the polymer solutions. The thermodynamics of the coacervation process can be explained in 3 steps by a ternary phase diagram at a specific heating/cooling step (Fig. 9C). First, referring to (Fig. 9C-I) after the preparation of the primary emulsion, the complete dissolution of the Ch in water is reached through the utilization of the AcOH. The working point is located outside of the miscibility gap that exists between water and Ch. In the second step (Fig. 9C-II), the Coacervation phase is added to the primary emulsion, promoting the dilution of the water phase: in this condition, the concentration of the AcOH solution decreases below the saturation limit. At the same time, keeping constant the Temperature, AcOH continues evaporating, inducing a further shifting of the working point in the miscibility gap, leading to the coacervation of HA on the chitosan template. In the last phase (Fig. 9C-III), a controlled cooling step is performed to increase the miscibility gap able to promote a faster supersaturation and the stability of the nanoparticle’s architecture. The obtained HyCoS NPs result stable and retain their cargo even after the conjugation of fluorescent molecules, making them particularly suitable for multimodal imaging applications. Indeed, the process conditions allow the formation of the core-shell structure while the entrapment of Gd-DTPA is reached through the crosslinking reactions. The proposed approach describes the successful conditions to obtain the production of HyCoS Nanoparticles with improved MRI properties. This effect can be explained according to the Solomon Bloemberg model^49^. Indeed, in this theory, the metal complex can be schemed as having separate coordination spheres, the Inner Sphere (IS) and the Outer Sphere (OS) both described by the characteristic correlation times, such as the residence time of the coordinated water molecule (τM), which in turn determines the rate of the coordinated water molecule exchanging with the bulk, and the rotational correlation time (τR), which is how quickly the contrast agent is tumbling in solution and the translational diffusional time (τD), which represent the diffusion of water molecules in the bulk near to the Gd complex^11^,^49^. Furthermore, we have recently reported that hydrogel matrices can strongly influence the relaxation rate of the Gadolinium-based Contrast Agents^11^,^49^. Because of our findings, we can assert that the improved MRI performances result from the double crosslinking able to control the entrapments of the Gd-DTPA at the Chitosan–HA interface and, therefore, to tune its physical characteristic parameters responsible for the relaxometric properties. The presented approach leverages the use of the coacervation kinetic and the crosslinking reaction, promoting the diffusion and exchange of the components at the interface HA-Ch and leading to a peculiar environment able to boost the relaxometric properties. The absence of these specific conditions will result in the absence of the MRI signal or even of the improved performances due to the hydrophobic nature of the Chitosan that would not allow the water exchange with the metal chelate, limiting its relaxivity.

## Conclusions

We obtained core-shell polymer NPs, which can be encapsulated with both Gd-DTPA and a Dye for Dual Imaging applications through a complex coacervation that exploits an innovative double crosslinking to improve the stability of the nanostructure overcoming the interference of the Gd-DTPA in the coacervation process. Furthermore, the adjustment of the process parameters, the coacervation and chemical reaction kinetic promote the interpolation of the hydrophobic core with the hydrophilic shell, controlling the water exchange and, consequently, the relaxation rate T_1_, enhancing the MRI signal at reduced concentration compared to the relevant clinical CAs. Furthermore, we individuated some process conditions able to develop a pH-sensitive behavior of the HyCoS NPs. However, further investigations are required to highlight the benefits and the drawbacks of this behavior and to consider the system as an effective and safe platform for theranostic nanomedicine. Future developments of this project are the upgrade of the designed nanosystem to the trimodal applications and *in vivo* tests. In particular, we aim to study our HyCoS NPs with the Fluorodeoxyglucose (^18^F-FDG) for Positron Emission Tomography (PET) and integrated MRI applications.

## Method

### Evaluation of the Phase behavior of the Ch-Acetic Acid-Water system

The behavior of the Ch- AcOH-water system is evaluated experimentally starting from 1 ml of water in which are added Ch and AcOH at different percentages alternatively. After each addition, the vial is stirred for 15 min. After the behavior of the Ch- AcOH-water system is observed, if the system resulted completely solved that point was indicated on the ternary diagram with a green spot, alternatively with a red one. The region, in which the behavior of the ternary system is only partially dissolved, is displayed with a black line to underline the difference between the two different behaviors.

### Emulsion preparation to produce core-shell nanoparticles by complex coacervation

A Complex Emulsion-Coacervation is used for the production of HyCoS nanostructures. The first step consists in the preparation of a w/o emulsion (5%/95% v/v) used as a template. The water phase is made of an aqueous solution of Ch and AcOH 8% v/v obtained by mixing 5 ml of MilliQ water at a Ch concentration ranging from 0.1% wt/v to 1% wt/v. Due to the hydrophobic nature of the Ch, the greater is the percentage of AcOH in the solution; the lower is the gap of miscibility between Ch and water. The oil phase is obtained by dissolving the surfactant Span80 (0.5–1% wt/v) in 45 ml of Mineral Oil and homogenizing for 5 min at 7000 rpm (by L5MA purchased by Silverton). The primary emulsion, obtained by mixing both phases, is treated for 20 minutes at 7000 rpm. Then, the solution containing HA, as a coacervate polymer, is added dropwise and the final volume is homogenized at 7000 rpm for further 30 min, keeping constant the Temperature at 37 °C. In a different preparation, a clinical relevant Contrast Agents (CAs), Gd-DTPA, at a concentration of 18 mM, is added to the aqueous solution containing Ch before mixing to the oil phase. When Gd-DTPA is added to the aqueous solution, the preferred volume of AcOH is of 10 μL to balance the acid nature of Gd-DTPA and to re-establish the pH conditions from 4.5 to 5 able to favor the activation of the –COOH group. The final concentration of Gd-DTPA within the total emulsion is of 1.8 mM. After the preparation of a stable primary emulsion, the coacervant phase, composed of 0.1% wt/v HA in a water solution of 3 ml at 30% wt/v, is added dropwise to the w/o emulsion template and homogenized at 7000 rpm for 30 min. The pH and temperature are continuously monitored and kept at a value ranging from 4.5–5. Coacervation starts as soon as HA reaches the surface of the droplet template containing the chitosan solution. To improve the stability of the coacervate, two crosslinking agents are added to the phases. The preferred formulation is the addition of TPP to the coacervant phase and DVS to the aqueous phase. TPP concentration ranges from 20–40%wt/v increasing the pH of the coacervant phase to 10–11. The addition of DVS (8–24%v/v) to the aqueous phase kept the pH condition at the constant value of 4.5–5. DVS is always added to the aqueous phase to avoid an uncontrolled crosslinking reaction. The total formulation is stirred overnight at 300 rpm at T ambient until coacervation in completely occurs. Time of homogenization is also studied by stirring the w/o emulsion for 5-10-15 or 20 min at 7000 rpm while the coacervation step is performed at 30 and 60 min at 7000 rpm. In different protocols, the effect of the Temperature is also tested and the coacervation step is maintained at high constant temperature for 6 h to promote the evaporation of the AcOH or a controlled Temperature profile is performed from 40 to 23 °C. To follow the development of the chemical reaction, few microliter of emulsion are collected at different time points and the formation of the nanoparticle is attempted by SEM.

### Evaluation of Encapsulation Efficiency and Loading Capability

To study the Encapsulation Efficiency (EE) and the Loading Capability (LC) of several specific dyes, Cy5, Atto 633, FITC, are alternatively added to the aqueous phase at concentrations ranging from 0.1 to 1 mg/mL. For the determination of the EE and LC the protocol proposed by Ankrum *et al*. is followed^50^. Furthermore, Induced Coupled Plasma (ICP-MS) - NexION 350 by Perkin Elmer is used to assess the concentration of the metal chelate in the nanoparticle suspension. Nanoparticles were suspended in a solution of deionized (DI) water at a concentration of 150.000 particles/mL. All data were collected and processed using the Syngistix Nano Application Module. Gd was measured at m/z 157 using a 100 μs dwell time with no settling time.

### Bioconjugation of the HyCoS NPs

230 μl of a solution of EDC at 70 mM and 230 μl of a solution of NHS at 21 mM are added to 500 μl of NPs to activate the carboxylic groups on the external shell of HA. After 10 min of stirring, 40 μl of the selected dye are added. The conjugation reaction is kept under continuous stirring for two hours at room temperature. Purification protocols are performed to wash away the residual reaction components by dialysis or ultracentrifugation. The behavior of the NPs is observed at two different pH values: 4.3 and 7. HCl and NaOH are added to reach the selected pH values. In particular, 8 μl of a solution 1 M of HCl are added to 5 ml of NPs to reach the pH of 4. Then, the suspension is split into 10 batches of 500 μl and kept under continuous stirring for 24 h. NPs, hold at pH 4, are observed at different time points, 30 min, 1 h, 2 h, 3 h, 6 h, 8 h, 12 h, 24 h by SEM. Alternatively, NPs are treated at pH 7, by adding 10 μl of a NaOH solution at 1 M to 5 ml of the aqueous phase containing NPs at pH value of 5.5. The observation of NPs at pH 7 is done after 30 min, 1 h, 2 h, 3 h, 6 h, 8 h, 12 h, 24 h at SEM. The experiments are repeated in triplicate.

### Characterization of HyCoS Nanoparticles

Dynamic light scattering (DLS) is used to determine nanoparticle size (Zeta sizer, Malvern UK). The wavelength of the laser is 633 nm and the scattering angle used is 173°. The cuvettes used are the 12 mm square glass couvettes for 90° sizing. An ideal sample submission for DLS analysis has a volume of 1–2 mL. In DLS analysis, the z-Average value represented the mean value of the hydrodynamic diameter of the particle, while the polydispersity index measured the width of the particle size distribution.

Zeta potential measurements are also performed at a temperature of 25 °C on a Zetasizer Nano ZS (Malvern, UK), fitted with a high-concentration zeta potential cell. Various concentrations were prepared to ensure that any differences in the measured electrophoretic mobilities were not due to changes in conductivity. Five repeat measurements on each sample were made to check the repeatability of the results obtained.

A Field Emission Scanning Electron Microscope (FE-SEM) by Zeiss, Transmission Electron Microscope (TEM) by FEI^®^ in DRY, CRYO and Tomography (TOMO) modes, Confocal Microscope (STED Stimulated Emission Depletion) and IR spectroscopy Thermo are used to characterize morphologically and chemically the system. The SEM characterizations are made after 48 h of stirring withdrawing 200 μl of the reaction and diluting in 50 ml of EtOH, after an ultracentrifugation (UC condition 15.000 rpm 10 min 4 °C) and after the dialysis to control the integrity of the nanostructures. The SEM observations are made by collecting nanoparticles on an ISOPORE membrane of 100 nm pore size. Nanoparticles are coated with 7 nm Au or PtPd prior observation. The TEM analyses are conducted both in DRY, CRYO and Tomography (TOMO) modes. In the DRY mode, the samples are prepared using Formvar/Carbon 200 mesh Cu Agar using 20 μl of the suspended nanoparticles. In CRYO mode the samples are prepared using VITROBOT FEI coating Lacey Carbon film 200 mesh Cu Agar with 3 μl of nanoparticles suspension. The conditions of VITROBOT are blotting time 1 s, humidity upper than 70% and temperature 20 °C. Confocal Microscopy (Stimulated Emission Depletion) STED is used to evaluate the fluorescence of encapsulated or conjugated NPs. The observed samples are prepared by drying 20 μl of nanoparticles suspension deposited in a FluorDish. FT-IR studies are conducted to investigate the interaction among Ch, TPP and HA in the nanoparticles formation process. The IR analysis are obtained by measuring solid samples of raw Chitosan, HA and TPP while 150 μl of HyCOs NPs are deposited on silicon wafers to avoid interference.

### *In Vitro* MRI

Analyses with Minispec 60 mq BRUKER are performed to evaluate the relaxation times. 300 μl of nanoparticles’ suspension or Gd-DTPA solution are loaded within a glass tube. The Free Induction Decay sequence (FID) is used to evaluate the best value of the Gain to control the saturation of the signal and measure and to measure T_2_^*^ signal. Also, the Saturation Recovery and Inversion Recovery sequences are used to measure T_1_ signal while the Carr-Purcell sequence (CPC) to evaluate T_2_. The relaxation time distribution are obtained by CONTIN Algorithm. The relaxation spectrum is normalized on the CONTIN processing parameters. The integral of a peak corresponds therefore to the contribution of the species exhibiting this peculiar relaxation to the relaxation time spectrum^51^. Experiments are repeated at least five times.

### *In vitro* cytotoxicity

An adenocarcinoma human alveolar basal epithelial cell line (A549) is used. The cells are cultured in Dulbecco’s modified Eagle’s medium (DMEM) containing 10% fetal calf serum and L-glutamine (2.9 mg/ml) at 37 °C in water-saturated air supplemented with 5% CO_2_. For the cytotoxicity measurements, the cells are plated in 96-well plates for 24 h before the addition of the nanoparticles. Fresh medium, containing an increasing concentration of nanoparticles, obtained at room temperature, is added to each well and the cells are incubated for 24 h. At the end of the incubation time, the cytotoxicity of the nanoparticles is tested using a WST-1 assay (SIGMA-ALDRICH, MI). Briefly, phosphate-buffered saline (PBS) containing WST-1 is added to each well, and the cells are incubated for 30 min, 1 h, 2 h, 4 h, 12 h and 24 h. Spectral absorption of the samples are measured at 450 nm with ELISA Reader (Biotek, Winooski, VT). The number of live cells is proportional to the amount of formazan produced.

## Additional Information

**How to cite this article:** Vecchione, D. *et al*. Hybrid Core-Shell Nanoparticles (HyCoS) produced by complex coacervation for Multimodal Applications. *Sci. Rep.* **7**, 45121; doi: 10.1038/srep45121 (2017).

**Publisher's note:** Springer Nature remains neutral with regard to jurisdictional claims in published maps and institutional affiliations.

## Supplementary Material

Supplementary Information

Supplementary Video 1

## References

[b1] Kyu T. & Saldanha J. M. Phase-separation by spinodal decomposition in polycarbonate poly(methyl methacrylate) blends. Macromolecules 21, 1021–1026, doi: 10.1021/ma00182a030 (1988).

[b2] Dowy S., Torino E., Luther S. K., Rossmann M. & Braeuer A. Imaging the supersaturation in high-pressure systems for particle generation. Chemical Engineering Journal 168, 896–902, doi: 10.1016/j.cej.2010.11.088 (2011).

[b3] Shao J. . Modified PLA Homochiral Crystallites Facilitated by the Confinement of PLA Stereocomplexes. Macromolecules 46, 6963–6971, doi: 10.1021/ma400938v (2013).

[b4] Esser-Kahn A. P., Odom S. A., Sottos N. R., White S. R. & Moore J. S. Triggered Release from Polymer Capsules. Macromolecules 44, 5539–5553, doi: 10.1021/ma201014n (2011).

[b5] McDonald R. J. . Gadolinium Deposition after Contrast-enhanced MR Imaging Response. Radiology 277, 925–925 (2015). 10.1148/radiol.15150025 25742194

[b6] Semelka R. C., Commander C. W., Jay M., Burke L. M. B. & Ramalho M. Presumed Gadolinium Toxicity in Subjects With Normal Renal Function: A Report of 4 Cases. Investigative Radiology 51, 661–665, doi: 10.1097/rli.0000000000000318 (2016). 27548344

[b7] Kartamihardja A. A. P., Nakajima T., Kameo S., Koyama H. & Tsushima Y. Impact of Impaired Renal Function on Gadolinium Retention After Administration of Gadolinium-Based Contrast Agents in a Mouse Model. Investigative Radiology 51, 655–660, doi: 10.1097/rli.0000000000000295 (2016). 27299580

[b8] Zhang L. . The evolution of gadolinium based contrast agents: from single-modality to multi-modality. Nanoscale 8, 10491–10510, doi: 10.1039/c6nr00267f (2016). 27159645

[b9] Cacciuto A. & Luijten E. Confinement-driven translocation of a flexible polymer. Physical Review Letters 96, doi: 10.1103/PhysRevLett.96.238104 (2006). 16803411

[b10] Mornet S., Vasseur S., Grasset F. & Duguet E. Magnetic nanoparticle design for medical diagnosis and therapy. Journal of Materials Chemistry 14, 2161–2175, doi: 10.1039/b402025a (2004).

[b11] Ponsiglione A. M., Russo M., Netti P. A. & Torino E. Impact of biopolymer matrices on relaxometric properties of contrast agents. Interface Focus 6, doi: 10.1098/rsfs.2016.0061 (2016). PMC5071819 27920897

[b12] Russo M., Bevilacqua P., Netti P. A. & Torino E. A Microfluidic Platform to design crosslinked Hyaluronic Acid Nanoparticles (cHANPs) for enhanced MRI. Scientific Reports 6, doi: 10.1038/srep37906 (2016). PMC5128828 27901092

[b13] Dudowicz J., Freed K. F. & Madden W. G. Role of molecular-structure on the thermodynamic properties of melts, blends, and concentrated polymer-solutions - comparison of monte-carlo simulations with the cluster theory for the lattice model. Macromolecules 23, 4803–4819, doi: 10.1021/ma00224a009 (1990).

[b14] Saito S. . Phase separation in a polymer solution induced by steady and large amplitude oscillatory shear flow. Macromolecules 36, 3745–3748, doi: 10.1021/ma0208584 (2003).

[b15] Fosnaric M., Iglic A., Kroll D. M. & May S. Monte Carlo simulations of a polymer confined within a fluid vesicle. Soft Matter 9, 3976–3984, doi: 10.1039/c3sm27938c (2013).

[b16] Flory P. J. Principles of Polymer Chemestry. (Cornell University, 1953).

[b17] Garlotta D. A literature review of poly(lactic acid). Journal of Polymers and the Environment 9, 63–84, doi: 10.1023/a:1020200822435 (2001).

[b18] Gizzatov A. . Geometrical confinement of Gd(DOTA) molecules within mesoporous silicon nanoconstructs for MR imaging of cancer. Cancer Letters 352, 97–101, doi: 10.1016/j.canlet.2014.06.001 (2014). 24931336 PMC4539146

[b19] Utech S. & Boccaccini A. R. A review of hydrogel-based composites for biomedical applications: enhancement of hydrogel properties by addition of rigid inorganic fillers. Journal of Materials Science 51, 271–310, doi: 10.1007/s10853-015-9382-5 (2016).

[b20] Xie J., Lee S. & Chen X. Y. Nanoparticle-based theranostic agents. Advanced Drug Delivery Reviews 62, 1064–1079, doi: 10.1016/j.addr.2010.07.009 (2010). 20691229 PMC2988080

[b21] Klymko K. & Cacciuto A. Free Energy of Multiple Overlapping Chains. Physical Review Letters 107, doi: 10.1103/PhysRevLett.107.278302 (2011). 22243330

[b22] Michels J. J. & Moons E. Simulation of Surface-Directed Phase Separation in a Solution-Processed Polymer/PCBM Blend. Macromolecules 46, 8693–8701, doi: 10.1021/ma400269j (2013).

[b23] Janib S. M., Moses A. S. & MacKay J. A. Imaging and drug delivery using theranostic nanoparticles. Advanced Drug Delivery Reviews 62, 1052–1063, doi: 10.1016/j.addr.2010.08.004 (2010). 20709124 PMC3769170

[b24] Blaker J. J., Knowles J. C. & Day R. M. Novel fabrication techniques to produce microspheres by thermally induced phase separation for tissue engineering and drug delivery. Acta Biomaterialia 4, 264–272, doi: 10.1016/j.actbio.2007.09.011 (2008). 18032120

[b25] Fomchenko E. I. & Holland E. C. Mouse models of brain tumors and their applications in preclinical trials. Clinical Cancer Research 12, 5288–5297, doi: 10.1158/1078-0432.ccr-06-0438 (2006). 17000661

[b26] Wang Y. T., Jiang Z. Y., Fu L. L., Lu Y. & Men Y. F. Stretching Temperature Dependency of Lamellar Thickness in Stress-Induced Localized Melting and Recrystallized Polybutene-1. Macromolecules 46, 7874–7879, doi: 10.1021/ma401326g (2013).

[b27] Koifman Khristosov M., Kabalah-Amitai L., Burghammer M., Katsman A. & Pokroy B. Formation of curved micrometer-sized single crystals. ACS nano 8, 4747–4753, doi: 10.1021/nn5013513 (2014). 24694217

[b28] Kubel C., Gonzalez-Ronda L., Drummy L. F. & Martin D. C. Defect-mediated curvature and twisting in polymer crystals. Journal of Physical Organic Chemistry 13, 816–829, doi: 10.1002/1099-1395(200012)13:12&lt;816::aid-poc322&gt;3.0.co;2-i (2000).

[b29] Potemkin, II . Spontaneous curvature of comblike polymers at a flat interface. Macromolecules 37, 3918–3923, doi: 10.1021/ma021519d (2004).

[b30] Khoury F. & Barnes D. Vol. 76A (Journal of Research of the Narional Bureau of Standards-A. Physics and Chemestry, 1972). 10.6028/jres.076A.027 PMC6706570 34565860

[b31] Mehta R., Keawwattana W., Guenthner A. L. & Kyu T. Role of curvature elasticity in sectorization and ripple formation during melt crystallization of polymer single crystals. Physical Review E 69, doi: 10.1103/PhysRevE.69.061802 (2004). 15244609

[b32] Cartier L. . Epitaxial crystallization and crystalline polymorphism of polylactides. Polymer 41, 8909–8919, doi: 10.1016/s0032-3861(00)00234-2 (2000).

[b33] Lee K. W. D., Chan P. K. & Feng X. S. A computational study of the polymerization-induced phase separation phenomenon in polymer solutions under a temperature gradient. Macromolecular Theory and Simulations 12, 413–424, doi: 10.1002/mats.200350003 (2003).

[b34] Nishitsuji S., Takenaka M. & Taniguchi T. Computer simulation study on the shear-induced phase separation in semi-dilute polymer solutions by using Ianniruberto-Marrucci model. Polymer 51, 1853–1860, doi: 10.1016/j.polymer.2010.02.031 (2010).

[b35] Rasouli G. & Rey A. D. Acoustic detection of pressure-induced phase separation spinodals in polymer solutions. Chemical Engineering Science 102, 67–75, doi: 10.1016/j.ces.2013.07.021 (2013).

[b36] Wasanasuk K. & Tashiro K. Structural Regularization in the Crystallization Process from the Glass or Melt of Poly(L-lactic Acid) Viewed from the Temperature-Dependent and Time-Resolved Measurements of FTIR and Wide-Angle/Small-Angle X-ray Scatterings. Macromolecules 44, 9650–9660, doi: 10.1021/ma2017666 (2011).

[b37] Kukadiya S. B., Chan P. K. & Mehrvar M. The Ludwig-Soret Effect on the Thermally Induced Phase Separation Process in Polymer Solutions: A Computational Study. Macromolecular Theory and Simulations 18, 97–107, doi: 10.1002/mats.200800074 (2009).

[b38] Li J., Rajagopalan R. & Jiang J. Polymer-induced phase separation and crystallization in immunoglobulin G solutions. Journal of Chemical Physics 128, doi: 10.1063/1.2919565 (2008). 18513048

[b39] Stylianopoulos T. & Jain R. K. Design considerations for nanotherapeutics in oncology. Nanomedicine: nanotechnology, biology, and medicine 11, 1893–1907, doi: 10.1016/j.nano.2015.07.015 (2015). PMC4628869 26282377

[b40] Jain R. K. & Stylianopoulos T. Delivering nanomedicine to solid tumors. Nature Reviews Clinical Oncology 7, 653–664, doi: 10.1038/nrclinonc.2010.139 (2010). PMC3065247 20838415

[b41] Adriana Martel-Estrada S., Alberto Martinez-Perez C., Guadalupe Chacon-Nava J., Elvia Garcia-Casillas P. & Olivas-Armendariz I. Synthesis and thermo-physical properties of chitosan/poly(DL-lactide-co-glycolide) composites prepared by thermally induced phase separation. Carbohydrate Polymers 81, 775–783, doi: 10.1016/j.carbpol.2010.03.032 (2010).

[b42] Collins M. N. & Birkinshaw C. Investigation of the swelling behavior of crosslinked hyaluronic acid films and hydrogels produced using homogeneous reactions. Journal of Applied Polymer Science 109, 923–931, doi: 10.1002/app.27631 (2008).

[b43] Reverchon E., De Marco I. & Torino E. Nanoparticles production by supercritical antisolvent precipitation: A general interpretation. The Journal of Supercritical Fluids 43, 126–138, doi: 10.1016/j.supflu.2007.04.013 (2007).

[b44] Reverchon E., Torino E., Dowy S., Braeuer A. & Leipertz A. Interactions of phase equilibria, jet fluid dynamics and mass transfer during supercritical antisolvent micronization. Chemical Engineering Journal 156, 446–458, doi: 10.1016/j.cej.2009.10.052 (2010).

[b45] Nguyen K. T. & Zhao Y. L. Engineered Hybrid Nanoparticles for On-Demand Diagnostics and Therapeutics. Accounts of Chemical Research 48, 3016–3025, doi: 10.1021/acs.accounts.5b00316 (2015). 26605438

[b46] Nasti A. . Chitosan/TPP and Chitosan/TPP-hyaluronic Acid Nanoparticles: Systematic Optimisation of the Preparative Process and Preliminary Biological Evaluation. Pharmaceutical Research 26, 1918–1930, doi: 10.1007/s11095-009-9908-0 (2009). 19507009

[b47] Soppimath K. S., Tan D. C. W. & Yang Y. Y. pH-triggered thermally responsive polymer core-shell nanoparticles for drug delivery. Advanced Materials 17, 318-+, doi: 10.1002/adma.200401057 (2005).

[b48] Papadimitriou S. A., Achilias D. S. & Bikiaris D. N. Chitosan-g-PEG nanoparticles ionically crosslinked with poly(glutamic acid) and tripolyphosphate as protein delivery systems. International Journal of Pharmaceutics 430, 318–327, doi: 10.1016/j.ijpharm.2012.04.004 (2012). 22521711

[b49] Caravan P. Strategies for increasing the sensitivity of gadolinium based MRI contrast agents. Chemical Society Reviews 35, 512–523, doi: 10.1039/b510982p (2006). 16729145

[b50] Ankrum J. A. . Engineering cells with intracellular agent-loaded microparticles to control cell phenotype. Nature Protocols 9, 233–245, doi: 10.1038/nprot.2014.002 (2014). 24407352 PMC4320648

[b51] Provencher S. W. CONTIN: a general purpose constrained regularization program for inverting noisy linear algebraic and integral equations. Computer Physics Communications 27, 229–242 (1982).

