# Supplementary material for: Hybrid Core-Shell (HyCoS) Nanoparticles produced by Complex Coacervation for Multimodal Applications
Source: Sci Rep. 2017 Mar 22;7:45121. doi: 10.1038/srep45121 (PMC5361191; doi:10.1038/srep45121)
Supplement: Supplementary Information [file srep45121-s1.pdf]

Supplementary Information

Hybrid Core-Shell (HyCoS) Nanoparticles produced by Complex Coacervation for Multimodal Applications

D. Vecchione[a,b], A. M. Grimaldi[c], E. Forte[c], Paolo Bevilacqua[c], P. A. Netti[a,b,d], E. Torino[a,d*].

[a] *Istituto Italiano di Tecnologia, Center for Advanced Biomaterials for Health Care IIT@CRIB, Largo Barsanti e Matteucci 53, 80125, Naples, Italy.*

[b] *University of Naples Federico II, Department of Chemical, Materials and Industrial Production Engineering, P.le Tecchio 80, 80125, Naples, Italy*

[c] *IRCSS SDN, Via E. Gianturco 113, 80143, Naples, Italy*

[d] *University of Naples Federico II, Interdisciplinary Research Center of Biomaterials, CRIB P.le Tecchio 80, 80125, Naples, Italy.*

*corresponding author: enza.torino@iit.it

Materials

Chitosan (Ch) low molecular weight; Divinyl Sulfone (DVS) 118.15 g/mol; Sodium Tripolyphosphate (TPP) 367.86 g/mol; Glacial Acetic Acid molecular weight 60.05; Ethanol (EtOH) molecular weight 46.07; Gd-DTPA molecular weight 547.57; Mineral Oil 0.84 g/mL at 25°C (lit.); Span80 molecular weight 274.43; 1.005 g/mL at 20 C (lit.); Dyes (Cy5, Atto 633, FITC) are purchased by Sigma-Aldrich® while Hyaluronic Acid (HA) 850 kDa parenteral grade is by Hyasis. MilliQ water is for all experiments.

Results

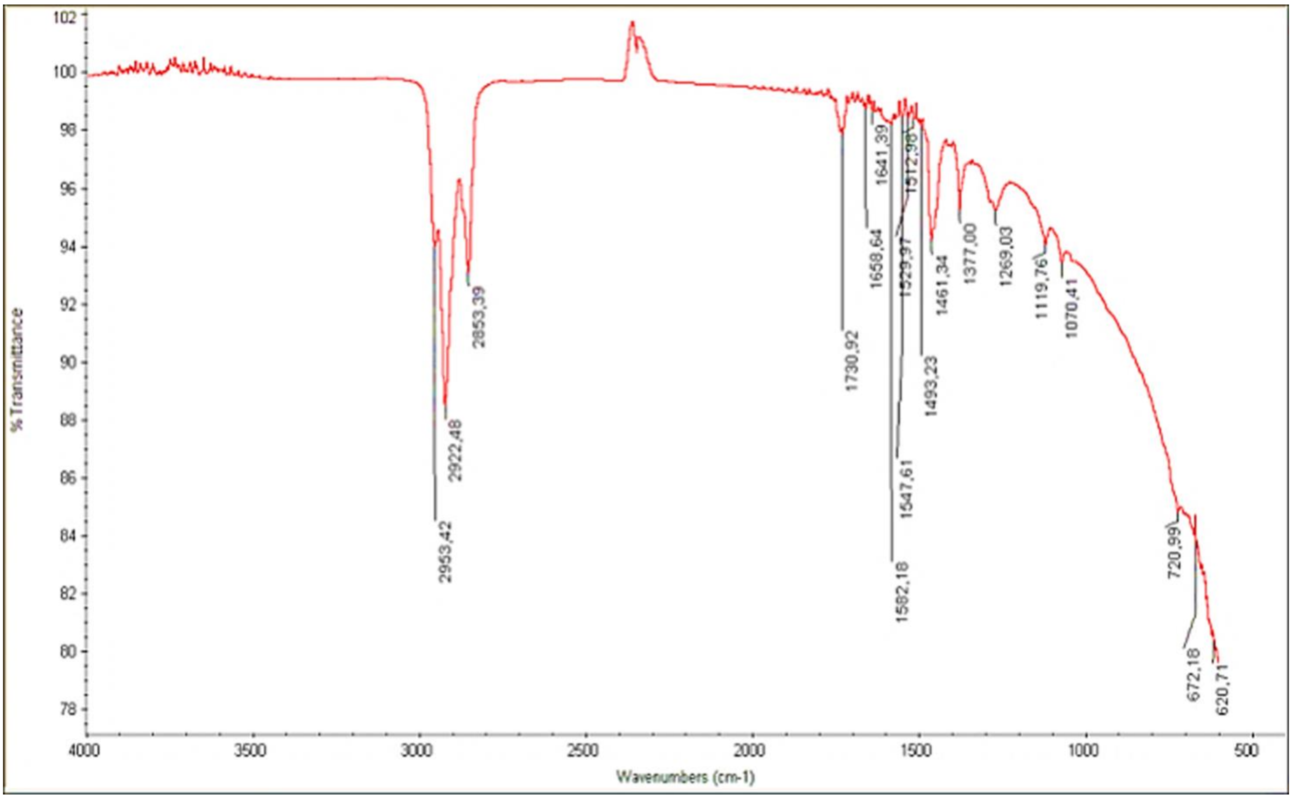

Figure S1: Linkage among the reactive groups analysed by FT-IR. The characteristic bands at 1658.64 cm-1 and 1730.92 cm-1 are attributed to C=O group stretching vibration in HA matrix. A new sharp peak 1641 cm-1 emerged and the 1.585 cm-1 peak of –NH2 bending vibration shifted to 1582 cm-1. Because of –NH2 bending vibration shifts, the characteristic bands at 1614 cm-1 and 1405 cm-1 shifted to 1641 cm-1 and 1377 cm-1 respectively. The presentation of P=O vibration absorption at 1269 cm-1 is observed, this indicates the reaction between CHS and TPP. The peaks at 2953.42 cm-1, 2922.48 cm-1 and 2853.39 cm-1 show the C-H interaction. The characteristic peaks for DVS exhibit absorption at 1119.76 cm-1 (S=O symmetric stretching vibrations) and 720.99 cm-1 (S−C stretching vibrations) and through the ether bond at 1269 cm-1 (C−O−C stretching vibrations).

Study of reaction times

The first part of this work is related to the study of the reaction times. When the final reaction is reached a demixing of the emulsion it can be observed and the formation of the nanoparticles results completed. Before this time, the coacervation reaction doesn`t occur, while after it, the particles begin to aggregate.

Study of homogenization times

The second part of this work is based on the study of the homogenization times. Leaving 5 min as a standard time for the homogenization between Oil and Span80, for the second and the third homogenization some different times are tested. For the w/o emulsion 5-10-15-20 min at 7000rpm are tested while for the third homogenization 30-60 min at 7000 rpm are tested. At the end, the best times are considered to be 20 min for w/o emulsion and 30 min for the final homogenization at 7000 rpm.

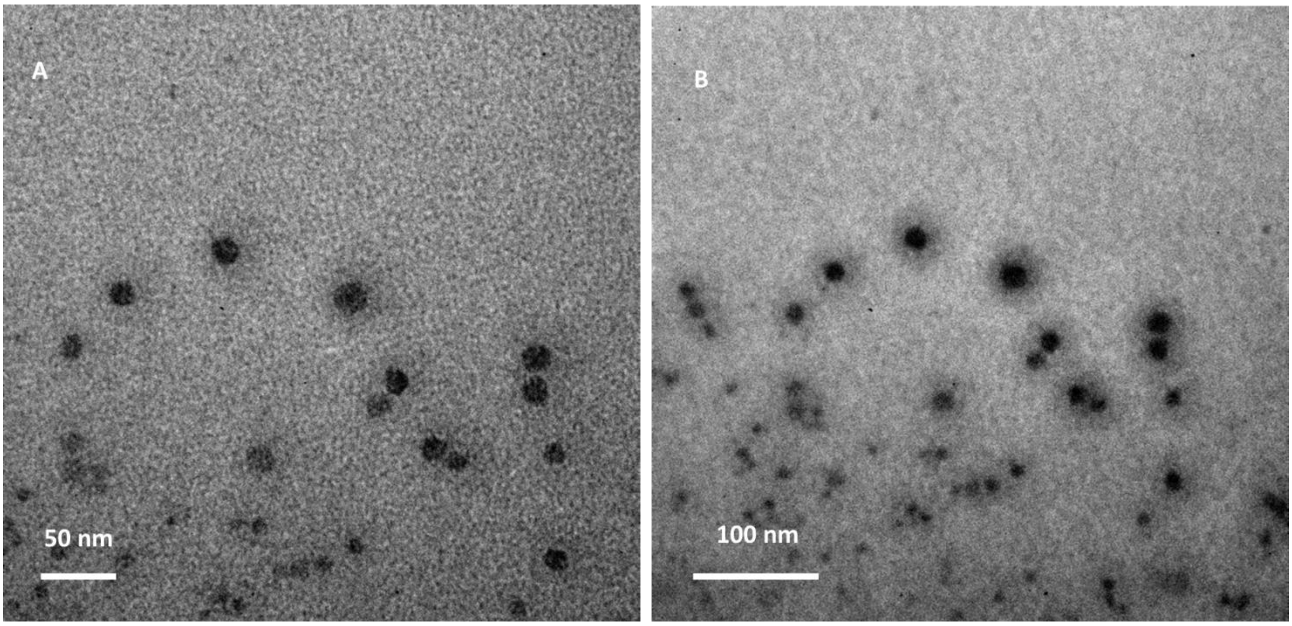

Figure S2: Gd-DTPA-Loaded HyCoS Nps obtained without performing a temperature profile and collected in water. (A and B at different scale bar) Nps obtained at 0,1% wt/v HA, 1% wt/v Chitosan, 30% TPP wt/v, Gd-DTPA: Chitosan 1:1 and 8% v/v DVS.

pH Study for the obtainment of a stable double crosslinking

A third study is conductedon the pH conditions. To allow the activation of the sites and the complete conclusion of the crosslinking reaction,all the prepared solutions are kept at specific pH values. A specific pH value (pH 5) is chosen as a good compromise between the activation of –COOH groups of HA and $–NH_3$ groups of chitosan[1]. The different percentage of the two crosslinkers are tested to investigate the swelling of the system. The tested percentage of TPP are 20%, 30% and 40% wt/v while 8%, 16% and 24% v/v for DVS.

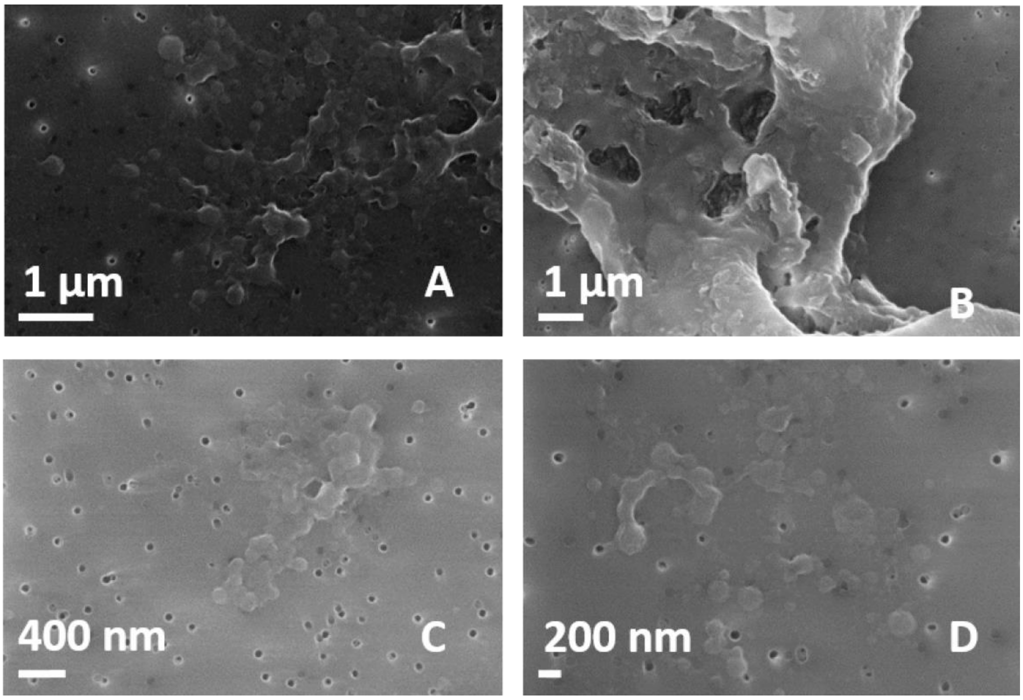

Figure S3: **<u>pH Responsive NPs</u>**

The behavior of the obtained NPs are tested at pH 4 and 7 at different time points: NPs mantained at pH 4 after 30 min (A); NPs mantained at pH 4 after 1h (B); NPs mantained at pH 7 after 30 min (C); NPs mantained at pH 7 after 24h (D).

[1] A. Nasti, N. M. Zaki, P. de Leonardis, S. Ungphaiboon, P. Sansongsak, M. G. Rimoli, N. Tirelli, Pharmaceutical Research 2009, 26, 1918; F. L. Mi, S. S. Shyu, S. T. Lee, T. B. Wong, Journal of Polymer Science Part B-Polymer Physics 1999, 37, 1551.

Video Legend

Video1: 3D reconstruction by cryo-TEM Tomography of Hybrid Core Shell Nanoparticles (HyCoS). Reconstruction is performed on HyCoS nanoparticles of about 200 nm to appreciate the tilting of the image and highlight the core-shell structures.
